# Quantifying Effects of Design Features on Youth Bicycle Helmet Performance During Oblique Impacts

**DOI:** 10.1007/s10439-025-03730-1

**Published:** 2025-04-05

**Authors:** Caitlyn Jung, Nicole E. -P. Stark, Susanna M. Gagliardi, Mark T. Begonia, Steve Rowson

**Affiliations:** 1 Department of Biomedical Engineering and Mechanics, Virginia Tech, 120 Kelly Hall, 325 Stanger Street MC 0298, Blacksburg, VA 24061 USA; 2 Institute for Critical Technology and Applied Science, Virginia Tech, Blacksburg, VA USA

**Keywords:** Helmets, Oblique impacts, Kids, Pediatric, Bike, Concussion

## Abstract

**Purpose:**

Cycling is a leading cause of youth sports-related head injury in the U.S. Although youth bicycle helmets sold in the U.S. comply with safety standards limiting head linear acceleration, there needs to be more information on relative differences in protection between helmets that pass. Additionally, studies have yet to look at quantifying youth bicycle helmet performance with respect to their design.

**Methods:**

Twenty-one youth bicycle helmet models were subjected to oblique impacts at three locations and two impact speeds where peak linear acceleration (PLA) and peak rotational acceleration (PRA) were quantified. Design features were characterized, including expanded polystyrene (EPS) thickness and presence of shell protrusions. A linear mixed model was used to quantify the effects of design features on PLA and PRA.

**Results:**

The youth bicycle helmet models evaluated produced wide ranges in kinematics across all configurations. PLA averaged 95.9 ± 26.1 g at 3.1 m/s and 170.1 ± 43.5 g at 5.2 m/s, while PRA averaged 3150 ± 1275 rad/s^2^ at 3.1 m/s and 4990 ± 1977 rad/s^2^ at 5.2 m/s. Impact location, impact speed, and EPS thickness had strong effects on PLA and PRA, whereas shell protrusions only had strong effects on PLA.

**Conclusion:**

Youth bicycle helmets with thicker EPS, thinner shells, and shell protrusions at impact locations improved the linear and rotational kinematic measures. Limitations include the small sample size and the impacts analyzed not representing all possible real-world scenarios.

## Introduction

In 2022, bicycle accidents were the fifth leading cause of concussion in children aged 14 and under [1]. However, this was based on emergency department visits to hospitals in the United States, so the actual prevalence of concussions is likely much higher. In 2020, bicycle accidents of children under 14 resulted in over 109,000 emergency room visits and 50 deaths nationwide, of which 26 were the result of traumatic brain injuries (TBIs) [2]. In 1994, Rivara et al. noted that implementing a community-wide bicycle helmet campaign decreased medically treated head injuries in 5- to 14-year olds by over 66% [3]. Now, there are 22 state-wide laws and over 200 localities with local ordinances for bicycle helmet use [4]. For 5- to 19-year-olds, helmet laws were associated with a 13% decrease in head injuries when comparing localities with bike helmet use ordinances to those without [5]. Helmets have also been shown to reduce head injury by 83.4% in pediatric bicyclists who are struck by motor vehicles [6].

The Consumer Product Safety Commission (CPSC) sets head injury safety standards to reduce the risk of catastrophic injury, such as skull fracture or TBI, and death by establishing limits for headform peak linear acceleration (PLA) metrics during helmet impact testing. Helmets marketed for both adults and children over age 5 must be tested in a series of guided drops with varying anvils and impact velocities and reduce PLA to less than 300 g [7]. This threshold was selected to prevent catastrophic head injury based on previous research indicating skull fracture occurs around a 300-g threshold [8–11]. However, concussions occur at much lower PLAs and result from linear and rotational accelerations [12–15], which certification standards do not account for; therefore, impact testing that evaluates rotational acceleration is important to better understand bicycle helmet performance.

While bicycle helmets marketed for children over age 5 and adults must meet the same safety standard, children and adults are thought to experience head injury at different biomechanical thresholds [12, 16, 17]. One study looking at concussion found that concussions in varsity football players (high school and collegiate level) occurred at 102 g on average, whereas in the youth population, the average concussive impact occurred at 62.4 g [12]. The difference in head acceleration exposures resulting in concussions culminated in different testing protocols for varsity and youth-level football [18, 19]. Similarly, it is expected that children impact their heads differently than adults due to lower ride height and traveling at slower speeds. Therefore, it is important to understand how youth bicycle helmets perform in youth-specific impact conditions to best understand their protective capabilities in this population. While there are currently testing protocols based on adult bicycle head impacts that provide information on helmet performance, there is a gap in research for youth bicycle helmet performance.

In a study by Hoshizaki et al., several youth helmets, including bicycle helmets, were tested to compare their protective abilities if used in tobogganing [20]. While this study did not focus on impact scenarios expected in youth bicycling and only evaluated one youth bicycling helmet, it showcased the helmet performance across various impact speeds when a monorail drop tower was used. Additionally, previous studies on adult bicycle helmet performance have shown ranges in protective performance with correlations between performance and various design characteristics, including style, shell thickness, and cost [21–25]. Youth bicycle helmets come in various styles, including road, urban, and full face. Some come with rotation-mitigating features, which intend to reduce rotational forces experienced by the head, thereby, lowering brain injury risk [26, 27]. Youth bicycle helmets also have more shell protrusions compared to adult bicycle helmets, such as animal faces and mohawks. These unique design features could alter the head’s kinematic response when engaged during impact.

This study aimed to relate youth bicycle helmets’ design features to their ability to reduce linear and rotational accelerations during youth-specific impact. We hope manufacturers consider this data when developing new helmet designs focused on reducing concussion risk.

## Materials and Methods

### Impact Testing

**Fig. 1:**
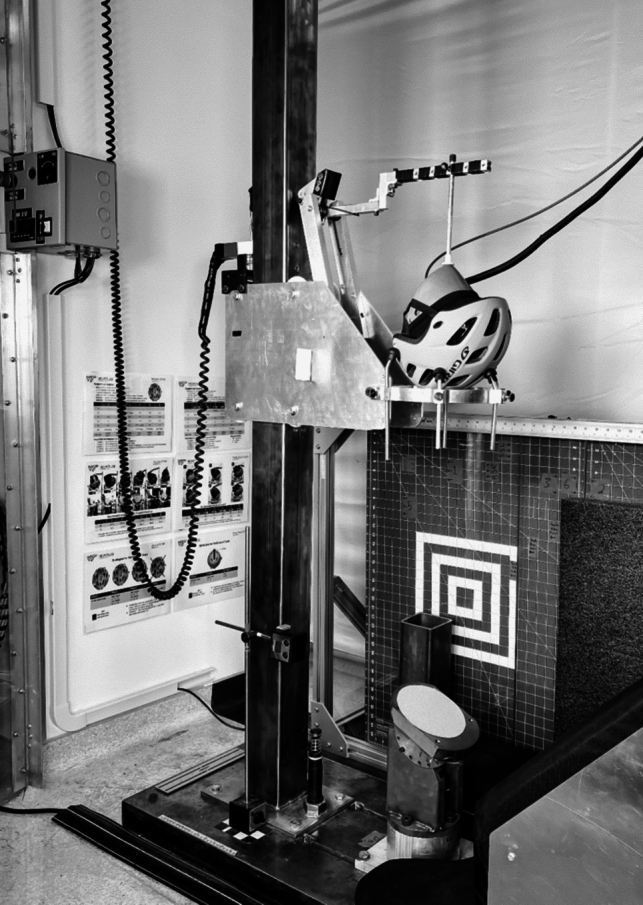
Oblique drop tower used for youth bicycle helmet testing. A helmeted headform is dropped onto an angled anvil to generate an oblique impact

An oblique drop tower was used for testing, where a helmeted 6-Year-Old Hybrid III headform (Humanetics, Farmington Hills, MI) was dropped onto a 25-degree steel anvil relative to horizontal (Fig. 1). 80-grit sandpaper was adhered to the anvil to simulate road-grit [25] and was replaced after every fourth test. Previous research by Stark et al. has noted that headforms have limitations in biofidelity, including their frictional characteristics [28, 29]. These studies noted that the Hybrid III headform with a skull cap has a coefficient of friction similar to that of the human head [29]. As such, a skull cap was used to reduce the friction between the headform and helmet to best match the frictional interface between the human head and helmet. The helmet was fitted on the headform using a custom tool whereby the lower front edge of the helmet was 13 cm from the chin, which is approximately 2 cm above the brow line [7]. The dial fit was adjusted until slight resistance could be detected, if applicable. The retention straps were tightened until nearly taut under the headform. The eye socket of the headform was used as a guide for centering each helmet relative to the midsagittal plane.

**Fig. 2:**
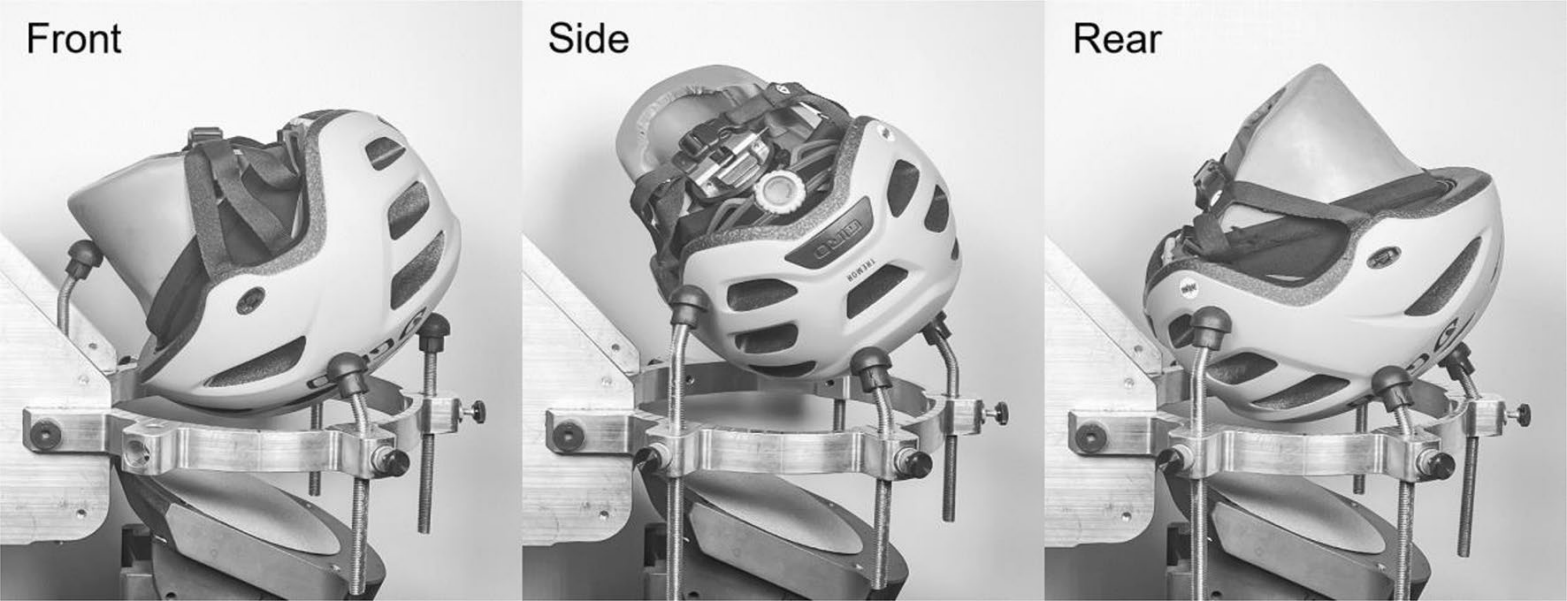
The three impact locations selected for testing and their orientation on the support ring

**Fig. 3:**
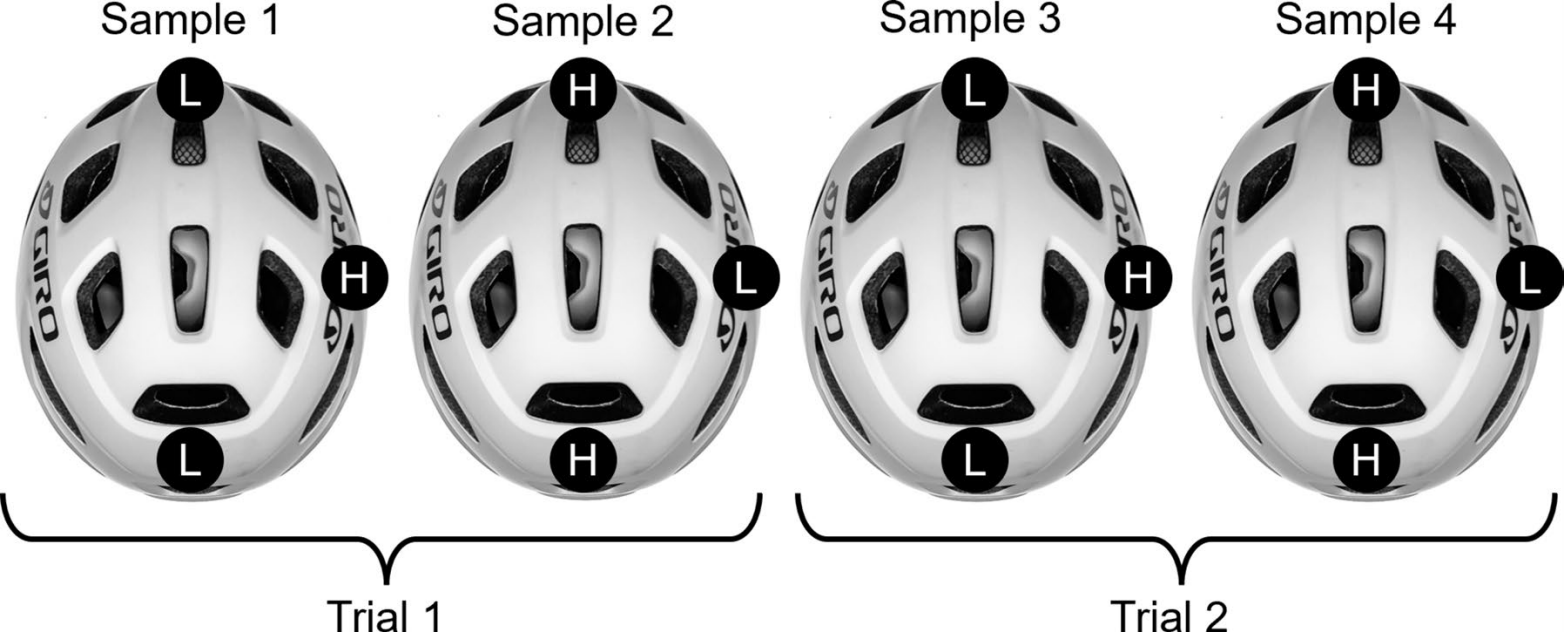
Impact configurations shown for the 4 samples used per helmet model. The superimposed circles represent the three impact locations, with L and H denoting low (3.1 m/s) and high (5.2 m/s) velocities, respectively. Two trials of each location-velocity combination are conducted

Impact conditions were selected to reflect those common in youth cyclist crashes as determined by publicly available videos on YouTube. In total, 24 videos were evaluated, of which 19 showed the individual impacting the front, 3 on the side, and 2 on the rear of their head. While these videos may not accurately represent the true ratio in which each location is impacted, they indicate variability in the impact locations. As such, three locations, front, side, and rear, were used as they are dispersed around the helmet, allowing us to assess performance over a range of impact scenarios (Fig. 2). Impact locations were set greater than 120 mm apart, as suggested by the CPSC standard, to prevent overlap of damage profiles from previous tests [7]. A dual-axis inclinometer, cross-level laser, and wall-mounted grid were used for positioning the headform orientation in the x-, y-, and z-orientation within the support ring to ensure consistency between trials. Impact velocities were based on percentages of maximum free fall normal speeds of an average 6-year-old standing height. The 60% energy condition represents the child moving slower and bracing before their head impacts and is associated with a normal speed of 2.85 m/s. The 100% energy condition represents the worst case scenario of a child hitting their head and is associated with a normal speed of 4.75 m/s. Standards are primarily based on normal velocities. However, during real-world impacts, it is unlikely that the head impacts a surface perpendicularly but instead at some oblique angle [24]. To maintain the normal velocities mentioned above, resultant velocities, which are combinations of normal and tangential velocities, must be transformed based on the impact angle due to the anvil angle. On the 25-degree anvil, these normal velocities were associated with resultant velocities of 3.1 and 5.2 m/s, which were used as target drop velocities (Table 1). Each helmet model is comprised of four helmet samples. Each helmet sample was impacted three times, only once per location at one of the two velocities (Fig. 3). Impact configurations are the combination of a single impact velocity (3.1 and 5.2 m/s) and impact location (front, side, rear). Two trials were conducted for each of the six impact configurations. This results in twelve tests per helmet model and 252 total tests across all twenty-one helmet models.

**Table 1:** Resultant velocities and their respective normal and tangential components

Resultant velocity (m/s)	Normal velocity (m/s)	Tangential velocity (m/s)
5.2	4.75	2.21
3.1	2.85	1.33

Linear and rotational head kinematics were recorded at 20 kHz during each test using a six degree-of-freedom sensor package, which consisted of three linear accelerometers (Endevco 7264B-2000, PCB Piezotronics, Charlotte, NC) and a tri-axis angular rate sensor (ARS3 PRO-18K, DTS, Seal Beach, CA) and was mounted at the headform center of the gravity. Data were filtered using a 4-pole phaseless Butterworth low pass filter in accordance with SAE J211 specifications [30]. We applied a cut-off frequency of 1650 Hz (channel frequency class [CFC] 1000) to accelerometer data and a cut-off frequency of 300 Hz (CFC 180) to angular rate sensor data. Rotational acceleration was computed using the 5-point central difference method in accordance with SAE J1727 specifications [31]. Resultant PLA and peak rotational acceleration (PRA) were used to estimate severity using a previously published equation specific for the youth population (Eq. 1) [12]. A log-normal cumulative distribution function (*µ* = 0.967 and *σ* = 0.331) was used to transform YGAMBIT (Eq. 1) to concussion risk. Cumulative concussion risk was determined by summing the individual risk values across the twelve impacts per helmet model, providing an overall risk value for each helmet model.

1 $$YGAMBIT=\sqrt{{\left(\frac{PLA}{62.4}\right)}^{2}+{\left(\frac{PRA}{2609}\right)}^{2}}.$$

### Helmet Model Design Features

**Fig. 4:**
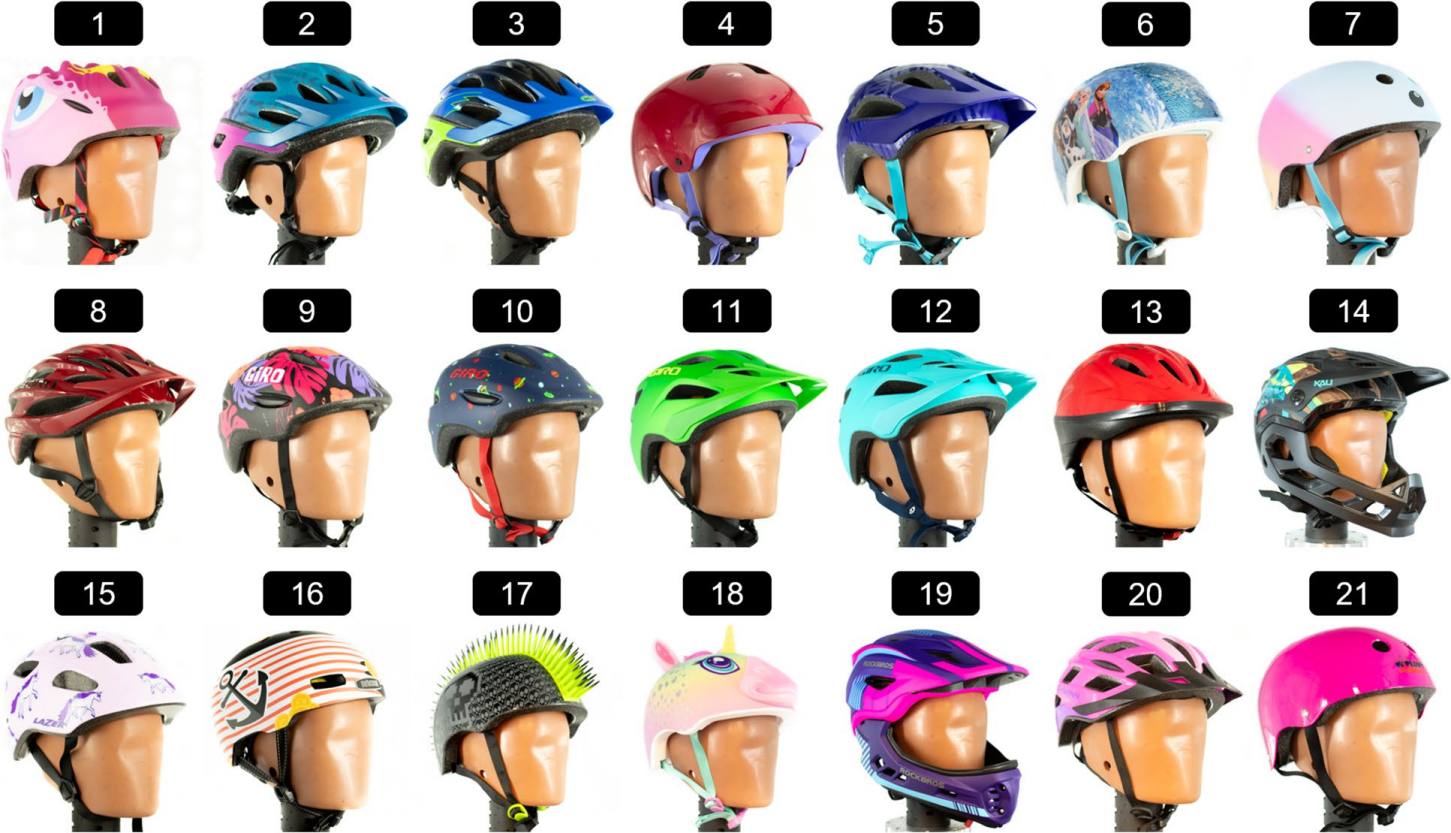
Helmet models selected for testing

Helmet models were selected by first researching which models were considered popular from internet searches. Additional models were added if they could be purchased from large, nationwide retailers. Because every youth bicycle helmet on the market cannot be tested, it was important to ensure we selected helmets that are widely available for parents to purchase both in store and online. Twenty-one CPSC-certified bicycle helmet models were purchased for testing (Fig. 4; Table 2), with sizes selected based on the Hybrid III 6-Year-Old head circumference. Fourteen brands were represented. The manufacturer suggested retail price (MSRP) at the time of purchase ranged from $25–140. Several helmet styles, including road, urban, full face, and those with shell protrusions, were incorporated to represent the multitude of styles available on the market. Several helmets contained rotational technology, including Multi-directional Impact Protection System (MIPS), WaveCel, and Kineticore. Rotational technology is intended to reduce the peak rotational head kinematics, but the mechanism by which they work varies between systems. MIPS is a low-friction layer mounted inside the helmet that is designed to move independently of the helmet in a crash [32]. Wavecel is a three-dimensional structure composed of a network of cells that intends to flex, crumple, or glide during a crash [33]. Kineticore is comprised of expanded polystyrene (EPS) blocks called controlled crumple zones that intend to absorb energy during a crash [34]. Visors were removed prior to testing, and internal helmet features were re-secured between tests when necessary.

**Table 2:** Helmet models selected for testing and their respective helmet style, presence of rotational technology, shell protrusions, shell finish, and manufacturer suggested retail price (MSRP) at the time of purchase

	Helmet model	Style	Rotational technology	Shell protrusions	Shell Finish	MSRP
1	Apusale Dinosaur	Road	None	None	Matte	$36
2	Bell Granite MIPS	Road	MIPS	None	Matte	$35
3	Bell Rev	Road	None	None	Glossy	$28
4	Bontrager Jet	Urban	WaveCel	None	Glossy	$53
5	Bontrager Tyro	Road	None	None	Glossy	$55
6	Disney Frozen Sequin	Road	None	None	Glossy and Sequins	$25
7	Eight Ball Helmet	Road	None	None	Matte	$25
8	Giro Raze	Road	None	None	Glossy	$50
9	Giro Scamp	Road	None	None	Matte	$41
10	Giro Scamp MIPS	Road	MIPS	None	Matte	$53
11	Giro Tremor	Road	None	None	Matte	$41
12	Giro Tremor MIPS	Road	MIPS	None	Matte	$55
13	Joovy Noodle	Road	None	None	Glossy	$30
14	Kali Maya Full Face	Full Face	None	None	Matte and Glossy	$140
15	Lazer Nutz	Road	Kineticore	None	Glossy	$50
16	Nutcase Little Nutty	Urban	MIPS	None	Matte	$70
17	Raskullz Flame Hawk	Road	None	Yes	Glossy	$28
18	Raskullz Super Rainbowcorn	Road	None	Yes	Matte and Glossy	$30
19	Rockbros Full Face	Full Face	None	None	Matte	$69
20	Schwinn Dash	Road	None	None	Glossy	$30
21	Triple8 Wipeout	Urban	None	None	Glossy	$30

Various design features were quantified, including EPS thickness, presence of shell protrusions, shell finish at each impact location, shell thickness, presence and type of rotational technology, and average helmet mass for each model (Tables 2, 3). EPS thickness was measured in mm in three locations within the area of impact center for each impact location using the caliper’s depth rod and averaged across location. Shell finish at each impact location was noted as matte, glossy, combination, or sequins. Shell thickness was measured in mm using calipers. Rotational technology was noted as MIPS, Wavecel, Kineticore, and None.

**Table 3:** Helmet models selected for testing and their respective average EPS thickness across all impact locations, shell thickness, and average mass

	Helmet model	EPS thickness (mm)	Shell thickness (mm)	Mass (grams)
1	Apusale Dinosaur	32.7	0.5	251
2	Bell Granite MIPS	27.5	0.5	283
3	Bell Rev	29.9	0.5	248
4	Bontrager Jet	26.8	2.5	585
5	Bontrager Tyro	29.6	0.5	303
6	Disney Frozen Sequin	26.9	0.5	250
7	Eight Ball Helmet	27.2	2.5	399
8	Giro Raze	25.1	0.5	262
9	Giro Scamp	26.4	0.5	235
10	Giro Scamp MIPS	26.1	0.5	250
11	Giro Tremor	26.7	0.5	228
12	Giro Tremor MIPS	27.5	0.5	249
13	Joovy Noodle	26.0	0.5	233
14	Kali Maya Full Face	24.5	0.5	524
15	Lazer Nutz	25.5	0.5	247
16	Nutcase Little Nutty	30.5	2.0	421
17	Raskullz Flame Hawk	26.1	0.5	319
18	Raskullz Super Rainbowcorn	27.4	0.5	328
19	Rockbros Full Face	33.0	0.5	420
20	Schwinn Dash	23.3	0.5	238
21	Triple8 Wipeout	26.2	2.3	349

### Statistical Analysis

We used R (Version 4.4.0, RStudio; Boston, MA) to evaluate PLA, PRA, and concussion risk using linear mixed-effect regression models (LMER) (lmerTest Package [35]), with a significance level *α* < 0.05. Separate LMER models compared impact location, impact speed, shell protrusions, shell finish, shell thickness, rotational technology, and EPS thickness, including helmet model as a random effect for PLA and PRA. Post hoc comparisons were completed using least squares means (lmerTest Package [35]) to compare pairwise differences for all features in the LMER model.

## Results

**Fig. 5:**
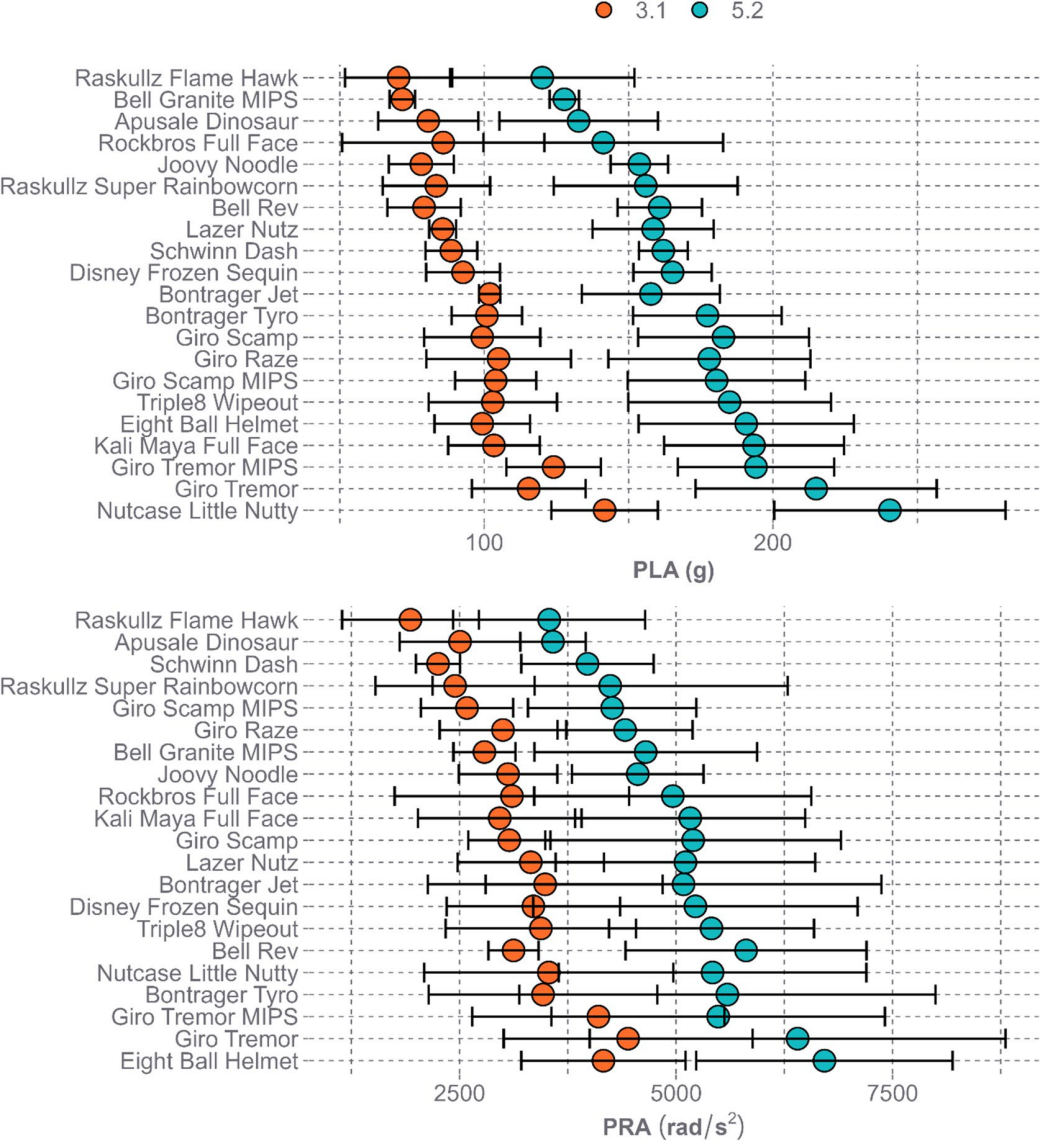
Mean PLA (top) and PRA (bottom) with 95% confidence intervals for each helmet model across all impact locations by impact speed

**Fig. 6:**
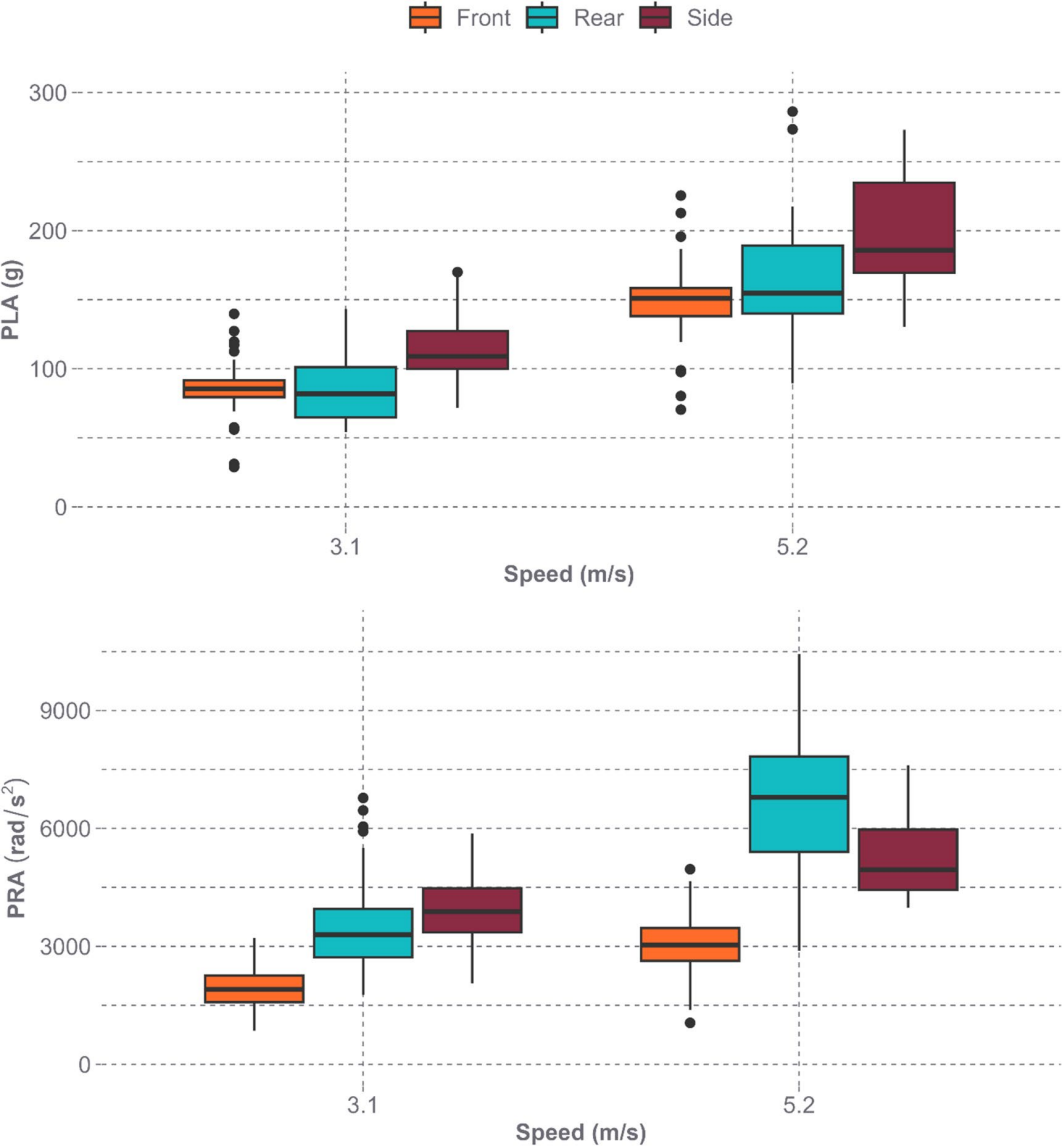
Distributions of PLA (top) and PRA (bottom) across helmet models by impact location and impact speed

PLA and PRA responses varied between the 21 helmet models (Fig. 5). At the low speed of 3.1 m/s, PLA values ranged from 28.8 to 170.0 g with an average of 95.9 ± 26.1 g, while PRA values ranged from 853 to 6776 rad/s^2^ with an average of 3150 ± 1275 rad/s^2^. At the high speed of 5.2 m/s, PLA values ranged from 70.4 to 286.2 g with an average of 170.1 ± 43.5 g, while PRA values ranged from 1052 to 10436 rad/s^2^ with an average of 4990 ± 1977 rad/s^2^. The front location was associated with lower PLAs and PRAs when compared to the side and rear locations at their respective speeds (Fig. 6).

**Fig. 7:**
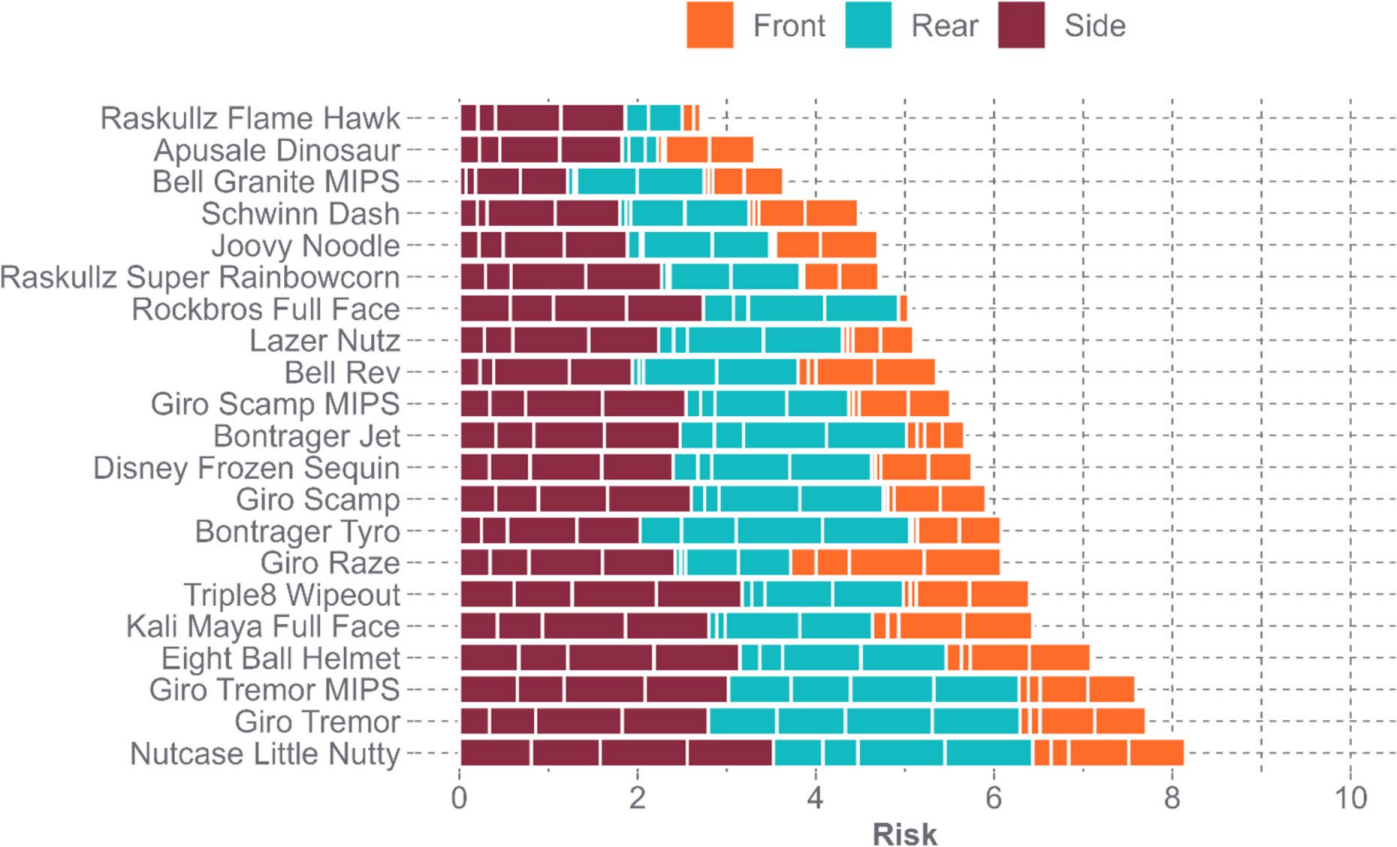
Cumulative concussion risk for each helmet model across all impact locations and impact speeds

Due to the range in PLA and PRA responses across helmet models, there was also a range in cumulative concussion risk values. Each impact can have a maximum concussion risk of 1.00, so across the helmet model, the cumulative concussion risk can be a maximum of 12.00. The cumulative concussion risk values for the helmet models ranged from 2.71 to 8.15, suggesting that the worst helmet had three times greater concussion risk compared to the best helmet (Fig. 7). There was an average risk of 5.59 ± 1.41.

The EPS thickness averaged 26.1 ± 5.0 mm, 25.2 ± 2.8 mm, and 30.8 ± 3.2 mm for the front, side, and rear impact locations, respectively. Two helmets had shell protrusions, both on the front and rear impact locations. Shell thickness averaged 0.85 ± 0.72 mm across all helmets and locations.

Impact location (*p* < 0.001) and impact speed (*p* < 0.001) had strong effects on PLA. For every additional mm of EPS thickness, PLA was reduced by 2.0 g (confidence interval [CI] 1.0–3.0 g, *p* < 0.001), while the presence of a shell protrusion at the site of impact reduced PLA by 24.6 g (CI 5.7–43.5 g, *p* = 0.011). However, for every additional mm of shell thickness, PLA was increased by 14.9 g (CI 0.2–29.6 g, *p* = 0.068).

When looking at PRA, impact location (*p* < 0.001) and impact speed (*p* < 0.001) continued to have strong effects. PRA was reduced by 44.8 rad/s^2^ (CI 2.0–87.6 rad/s^2^, *p* = 0.042) for every additional mm of EPS thickness, while for every additional mm of shell thickness, PRA increased by 465.5 rad/s^2^ (CI -18.1–949.1 rad/s^2^, *p* = 0.081). Shell protrusions at the site of impact reduced PRA by 542.1 rad/s^2^ (CI -260.2–1344.3 rad/s^2^, *p* = 0.187) when compared to no protrusion. The shell finish at the site of impact (*p* = 0.436) and the presence of rotational technology (*p* = 0.758) had no evidence of an effect on PRA.

## Discussion

**Fig. 8:**
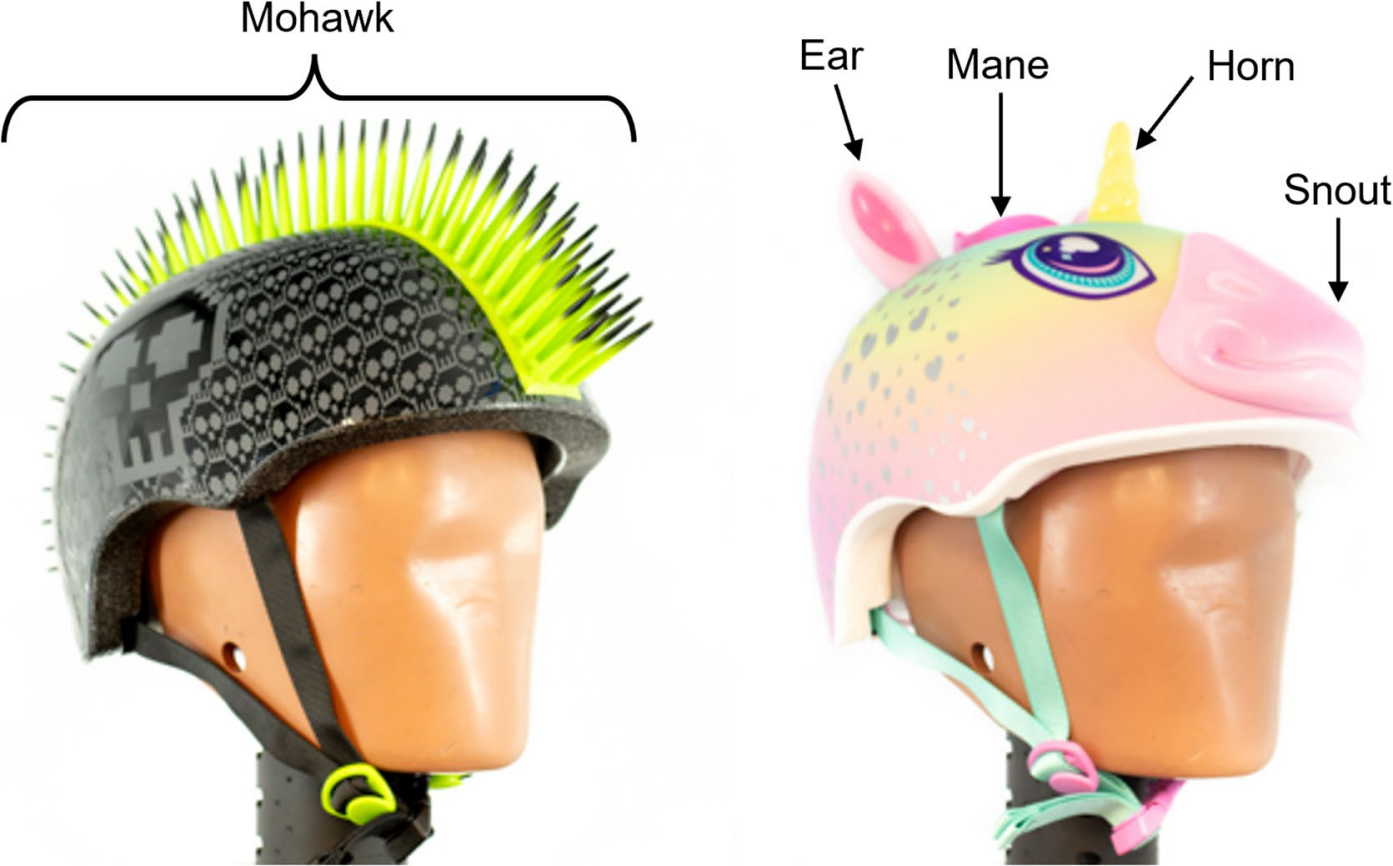
The youth bicycle helmets tested that had shell protrusions. The Raskullz Flame Hawk (left) had a mohawk TPR protrusion, while the Raskullz Super Rainbowcorn (right) had TPR protrusions to mimic an animal face consisting of a snout, horn, ears, and mane

The purpose of our study was to evaluate how 21 commercially available youth bicycle helmets performed during oblique impacts with respect to their design features. While all helmet models tested kept their PLA response below the 300 g standard set forth by CPSC, there was a range in performance in PLA, PRA, and concussion risk across the helmet models. Of the bicycle helmet models tested, Raskullz Flame Hawk reduced PLA, PRA, and concussion risk the most (Figs. 5 and 7). This helmet model had average EPS thickness and a thinner shell thickness compared to other models tested, as well as protrusions at two impact locations (Fig. 8). The Nutcase Little Nutty reduced PLA the least, while Eight Ball Helmet reduced PRA the least (Fig. 5), with the Nutcase Little Nutty resulting in the largest cumulative concussion risk (Fig. 7). Both Nutcase Little Nutty and Eight Ball Helmet had above average EPS thickness; however, they both had thicker shells than average. The Nutcase Little Nutty had triple the cumulative concussion risk of the Raskullz Flame Hawk helmet. This could be attributed to the various design characteristics that differ between each helmet model.

Multiple studies on adult bicycle helmet performance found a range in PLA performance across helmet models. Bland et al.’s 7.3-m/s oblique impact tests resulted in PLAs between approximately 100 and 275 g, with an average of 183.4 g [25]. This results in a standardized range (defined as range/mean) of 95%. Two other studies, Stigson et al. and Deck et al., both completed testing at 6.0 m/s onto a 45-degree anvil [21, 22]. Stigson et al. found PLA ranged from 74 to 180 g when excluding the Hovding 2.0, an airbag style helmet, and an average of 106 g resulting in a standardized range of 84%. Deck et al. found PLAs ranged from approximately 80 to 190 g. Baker et al. completed testing at 6.5 m/s onto a 45-degree anvil and found PLA ranged from 79.7 to 213.3 g across all locations and helmet models tested with an average of 133.6 g [23]. This results in a standardized range of 93%. The one study to have tested a youth bicycle helmet during toboggin-based impact conditions found average PLAs of 89.7 ± 5.3 g at the front location and 97.8 ± 7.6 g at the side location at 4.0 m/s and 128.1 ± 5.4 g at the front location and 166.0 ± 17.2 g at the side location at 6.0 m/s, using a monorail drop tower [20]. Our study found a slightly higher range in average PLAs across impact locations at the 5.2-m/s impact speed for youth bicycling helmets with a range of 120 to 240 g (Fig. 5) and average of 170.1 g compared to the adult bicycle helmet testing and the one youth bike helmet tested in a previous study. However, our study had a lower standardized range, 71%, than previous studies indicating helmets performed more similarly across the study than in previous studies. It is important to note that our study used a smaller headform and lower normal speed, so differences are to be expected.

PRA performance across adult bicycle helmets also varied across previous studies. Stigson et al.’s oblique impact tests resulted in PRAs ranging from 4200 to 18100 rad/s^2^ when excluding the Hovding 2.0 helmet [21]. The tests completed by Deck et al. found PRAs ranged from approximately 2000 to 12000 rad/s^2^ and an average of 8741 rad/s^2^ resulting in a standardized range of 159% [22]. Baker et al. found PRAs ranged from 1600 to 9700 rad/s^2^ and an average of 5273 rad/s^2^ resulting in a standardized range of 155% [23]. The one youth bicycle helmet study found average PRAs of 5448 ± 293 rad/s^2^ at the front location and 8158 ± 1212 rad/s^2^ at the side location at 4.0 m/s and 2198 ± 152 rad/s^2^ at the front location and 3684 ± 547 rad/s^2^ at the side location at 6.0 m/s [20]. Our study found PRAs across impact locations at the 5.2-m/s impact speed for youth bicycling helmets ranged from 3534 to 6713 rad/s^2^ (Fig. 5) and an average of 4990 rad/s^2^ to be in line with the PRAs found in adult bicycle helmet testing and the one youth bike helmet tested in a previous study. Similar to PLA, our study again noted a lower standardized range, 64%, than previous studies. It is expected that the differences between studies are in part due to the variation in anvil angle between studies.

Several design features affected the performance of youth bicycle helmets. First, EPS thickness had a strong impact on the linear and rotational acceleration measures. For every additional mm of EPS thickness, PLA was reduced by 2.0 g and PRA by 44.8 rad/s^2^. When looking at the PLAs and PRAs recorded in this study, these are not large reductions; however, they are very consistent between helmet models. As the EPS increased in thickness, the accelerations decreased because a thicker liner can be less stiff and deform more during impact.

Second, there was some evidence of shell thickness affecting all measures; thicker shells were associated with higher measures. PLA was increased by 14.9 g and PRA by 465.5 rad/s^2^ for every additional mm of shell thickness. These were large additions in accelerations; however, there was high variability between helmet models. Of the 21 helmet models tested, four had shell thicknesses of at least 2.0 mm, whereas the remaining helmet models had thicknesses of 0.5 mm. This resulted in wide confidence intervals when looking at shell thickness impact on the linear and rotational measures. Thicker shells are generally stiffer, which engages the EPS liner more, thus increasing the loading area on the EPS, which increases the effective stiffness of the foam. Of the four helmets with the thickest shells, three were dual-certified between CPSC for bicycle helmets and the American Society for Testing and Materials (ASTM) for skateboarding helmets [36]. In this standard, the helmet must be tested in a series of guided drops with varying anvils with impact velocities of 4.57 m/s and reduce PLA to less than 300 g, similar to the CPSC standard for bike helmets. However, ASTM testing for skateboarding helmets has an additional test which requires a helmet to be dropped three consecutive times at the same test location to evaluate the helmet’s ability to withstand multiple impacts. The goal of passing two different certification standards, CPSC for bike helmets and ASTM for skateboarding helmets, could have contributed to the design choice of having a thicker shell to pass the multiple impact condition in the ASTM standard.

Third, the presence of protrusions on the helmet’s exterior at the impact location improved the helmet’s ability to reduce acceleration. The helmets in our study had protrusions constructed of thermoplastic rubber (TPR), which is a deformable material, in two different designs (Fig. 8). There was a clear effect on PLA, but a wide confidence interval for PRA. While the protrusions are made of the same material across both helmet models, their placements on the headform and geometries of the protrusions themselves vary. These differences in placement and geometry could have resulted in differences in rotational kinematic response and therefore explain a higher confidence interval when looking at PRA. Helmet protrusions are suspected to aid in reducing PLA by acting similarly to the crumple zone of a car because the protrusions can deform and dissipate linear acceleration before engaging the EPS liner to continue decreasing the energy experienced in the headform. However, further research needs to be conducted to fully understand their role on PLA and PRA as the specific design of the TPR, such as mohawk vs animal face, could result in snagging or force concentrating in one location, which could result in greater acceleration and higher concussion risk depending on the impact conditions they are tested in. However, in the current data, the protrusions seem beneficial by reducing head kinematics.

Rotational technologies did not have an impact on PRA in our study. Several rotational technologies were tested in our study including MIPS, Wavecel, and Kineticore. In a study by Bliven et al., Wavecel and MIPS were compared in adult bicycle helmets over a range of anvil angles and speeds against an anvil covered in 80-grit sandpaper and found that Wavecel reduced rotational acceleration between 34 and 73%, while MIPS reduced rotational acceleration between 21 and 44% when compared to the control condition [26]. Bonin et al. noted that adding MIPS to adult bicycle helmets reduced peak angular acceleration by 38.2% across all headform conditions when completing tests on a 45-degree anvil with 40-grit sandpaper [27]. However, our study showed no evidence of these technologies reducing the rotational accelerations experienced by the headform. Rotational technologies may be optimized for impacts on a 45-degree anvil with 80-grit sandpaper, which could result in greater rotational acceleration of a headform during an oblique impact. This test configuration may allow these technologies to demonstrate their rotation mitigation capabilities under ideal impact conditions. In contrast, our study—even when using matched drop heights in previous studies—would generate lower tangential forces due to the shallower anvil angle and smaller headform mass. The range of tangential forces these technologies were designed for remains unclear, but our study likely produced lower tangential forces than previous studies that highlighted their effectiveness. This difference is attributed to the combination of the 25-degree anvil, lower drop heights, and the smaller headform mass representing a child, which may not have notably influenced PRA. In our study, a total of six helmets had rotational technology (4 MIPS, 1 Wavecel, 1 Kineticore); it is possible that the low sample size did not have enough power to find a notable difference.

Finally, shell finish did not have an impact on PLA or PRA in our study. Previous work by Stark et al. found the shell finish to be important on lower friction anvils [37]. In the same study, similar coefficients of friction between helmets with a glossy versus matte shell finish when tested against 80-grit sandpaper. Because our study also used 80-grit sandpaper on the anvil, we do not suspect that the shell finish at the impact location had an impact on the head kinematics.

Our study had several limitations. First, only 21 youth bicycle helmet models were chosen for testing. Models were chosen from a range of common retailers to represent a range of manufacturers, costs, and styles, but not all models currently on the market were tested. The results of this study are dependent on the design features of the selected helmets. Therefore, differing the models selected or altering the number of helmets that have specific design features could result in design features having greater or lesser importance than what was found in this study. With that being said, there were some design feature categories with a low number of samples, and that may have understated the significance of those design features. We recommend future work that further analyzes the design features in this study and how they impact concussion risk. Second, there may be some real-world impact scenarios that were not represented by the laboratory helmet testing protocol used in our study. The impact conditions selected may not be generalizable to the head impacts in the real world as the impact conditions were based on videos found online. These videos could have biased the impact conditions to ones that represent specific impact surfaces, rider age, gender, or those considered funny for viewer engagement rather than those that occur most often in youth bicyclists. This bias could lead to issues in generalizing the impacts tested in the lab to those happening in the real world, which may alter the performance capabilities of the youth bicycle helmets in a real-world setting compared to in the lab. While this study does not have a sample that completely represents the population, the protocol was considered robust, because it included three helmet impact locations and two impact speeds that allowed for a range in impact performance to be captured. Third, the impact locations and speeds were approximated but not quantified with software from the publicly available videos. Future work could explore the head impact kinematics experienced in the real world by youth bicyclists. Ideally, head kinematic data could be collected using the real-time sensor technologies or through high-speed video analysis of head impacts occurring in the real world. In addition to the kinematic data, having laboratory reconstructions of youth bicycle helmets that have been damaged in real-world head impacts would allow for further research into which accelerations and locations are common impact scenarios in youth bicyclists. Similar work has been completed when evaluating adult head impacts in bicycling [38]. Other further investigations should look at the role shell protrusions play in reducing acceleration. Only two helmets in our study had shell protrusions, and many other designs exist on the market. Further research needs to be conducted to determine how they may affect other impact conditions and whether they snag or localize force depending on the design of the shell protrusion.

Based on the results from our study, we find it important for youth bicycle helmet manufacturers to consider both linear and rotational head kinematics in lab assessments of youth bicycle helmet performance. This will improve the protective capabilities of the helmets beyond those that can be determined by CPSC standards testing.

Our study investigated the performance of 21 commercially available youth bicycle helmets with a helmet testing protocol that mimics impact conditions seen in publicly available youth bicycling head impact videos. These helmets were evaluated under oblique impacts to quantify the linear and rotational head kinematics of wearing a youth bicycle helmet. The head kinematics were used to determine cumulative concussion risk, which varied widely across helmet models. Our study was a comprehensive laboratory-based assessment of multiple youth bicycling helmet models that are currently available to consumers, but it is important to note that the reductions in accelerations are specific to the impact conditions used in the testing protocol. As not everyone experiences these same impacts, reductions in accelerations will vary from the averages presented here.

## References

[1] “CPSC NEISS On-Line Query System,” U.S. Consumer Product Safety Commission. Accessed: Oct. 18, 2023. [Online]. Available: https://www.cpsc.gov/cgibin/NEISSQuery/home.aspx

[2] “WISQARS Fatal and Nonfatal Injury Reports,” Centers for Disease Control and Prevention. Accessed: Oct. 18, 2023. [Online]. Available: https://wisqars.cdc.gov/reports/

[3] Rivara, F. P., et al. The Seattle children’s bicycle helmet campaign: changes in helmet use and head injury admissions. *Pediatrics*. 93(4):567–569, 1994. 8134210

[4] “Bicycle Helmet Laws.” Accessed: Nov. 11, 2024. [Online]. Available: https://helmets.org/mandator.htm

[5] Markowitz, S., and P. Chatterji. Effects of bicycle helmet laws on children’s injuries. *Health Econ*. 24(1):26–40, 2015. 10.1002/hec.2997. 24115375 10.1002/hec.2997

[6] Strotmeyer, S. J., C. Behr, A. Fabio, and B. A. Gaines. Bike helmets prevent pediatric head injury in serious bicycle crashes with motor vehicles. *Inj. Epidemiol.* 7(S1):24, 2020. 10.1186/s40621-020-00249-y. 32532330 10.1186/s40621-020-00249-y PMC7291179

[7] Safety Standard for Bicycle Helmets Final Rule (16 CFR Part 1203), 1998. Accessed: Oct. 16, 2023. [Online]. Available: https://www.ecfr.gov/current/title-16/part-1203

[8] Post, A., T. B. Hoshizaki, M. D. Gilchrist, S. Brien, M. Cusimano, and S. Marshall. The dynamic response characteristics of traumatic brain injury. *Accid. Anal. Prev.* 79:33–40, 2015. 10.1016/j.aap.2015.03.017. 25795923 10.1016/j.aap.2015.03.017

[9] Cachau-Hansgardh, A., C. McCleery, M. Limousis-Gayda, and R. Hashish. Analysis of bicycle helmet damage visibility for concussion-threshold impacts. *Int. Biomech.* 8(1):85–100, 2021. 10.1080/23335432.2021.2014359. 34915815 10.1080/23335432.2021.2014359 PMC8735878

[10] Mertz, H. J., P. Prasad, and A. L. Irwin, “Injury risk curves for children and adults in frontal and rear collisions,” presented at the 41st Stapp Car Crash Conference, Nov. 1997, p. 973318. 10.4271/973318.

[11] Yoganandan, N., and F. A. Pintar. Biomechanics of temporo-parietal skull fracture. *Clin. Biomech.* 19(3):225–239, 2004. 10.1016/j.clinbiomech.2003.12.014. 10.1016/j.clinbiomech.2003.12.014 15003337

[12] Campolettano, E. T., et al. Development of a concussion risk function for a youth population using head linear and rotational acceleration. *Ann. Biomed. Eng.* 48(1):92–103, 2020. 10.1007/s10439-019-02382-2. 31659605 10.1007/s10439-019-02382-2 PMC6928097

[13] Meaney, D. F., and D. H. Smith. Biomechanics of concussion. *Clin. Sports Med.* 30(1):19–31, 2011. 10.1016/j.csm.2010.08.009. 21074079 10.1016/j.csm.2010.08.009 PMC3979340

[14] Kleiven, S. Why most traumatic brain injuries are not caused by linear acceleration but skull fractures are. *Front. Bioeng. Biotechnol.* 1:15, 2013. 10.3389/fbioe.2013.00015. 25022321 10.3389/fbioe.2013.00015 PMC4090913

[15] Rowson, S., et al. Rotational head kinematics in football impacts: an injury risk function for concussion. *Ann. Biomed. Eng.* 40(1):1–13, 2012. 10.1007/s10439-011-0392-4. 22012081 10.1007/s10439-011-0392-4 PMC10465647

[16] Figaji, A. A. Anatomical and physiological differences between children and adults relevant to traumatic brain injury and the implications for clinical assessment and care. *Front. Neurol.* 2017. 10.3389/fneur.2017.00685. 29312119 10.3389/fneur.2017.00685 PMC5735372

[17] Margulies, S., and B. Coats. Experimental injury biomechanics of the pediatric head and brain. In: Pediatric injury biomechanics: archive & textbook, edited by J. R. Crandall, B. S. Myers, D. F. Meaney, and S. Zellers Schmidtke. New York: Springer, 2013, pp. 157–189.

[18] Rowson, S., and S. M. Duma. Development of the STAR evaluation system for football helmets: integrating player head impact exposure and risk of concussion. *Ann. Biomed. Eng.* 39(8):2130–2140, 2011. 10.1007/s10439-011-0322-5. 21553135 10.1007/s10439-011-0322-5

[19] Campolettano, E. T., R. A. Gellner, D. W. Sproule, M. T. Begonia, and S. Rowson. Quantifying youth football helmet performance: assessing linear and rotational head acceleration. *Ann. Biomed. Eng.* 48(6):1640–1650, 2020. 10.1007/s10439-020-02505-0. 32266597 10.1007/s10439-020-02505-0 PMC7494015

[20] Hoshizaki, B., M. Vassilyadi, A. Post, and A. Oeur. Performance analysis of winter activity protection headgear for young children. *J. Neurosurg. Pediatr.* 9(2):133–138, 2012. 10.3171/2011.11.PEDS11299. 22295916 10.3171/2011.11.PEDS11299

[21] Stigson, H., M. Rizzi, A. Ydenius, E. Engström, and A. Kullgren, “Consumer Testing of Bicycle Helmets,” 2017.

[22] Deck, C., N. Bourdet, F. Meyer, and R. Willinger. Protection performance of bicycle helmets. *J. Saf. Res.* 71:67–77, 2019. 10.1016/j.jsr.2019.09.003. 10.1016/j.jsr.2019.09.003 31862046

[23] Baker, C. E., X. Yu, B. Lovell, R. Tan, S. Patel, and M. Ghajari. How well do popular bicycle helmets protect from different types of head injury? *Ann. Biomed. Eng.* 2024. 10.1007/s10439-024-03589-8. 39294466 10.1007/s10439-024-03589-8 PMC11561050

[24] Bland, M. L., C. McNally, and S. Rowson. Differences in impact performance of bicycle helmets during oblique impacts. *J. Biomech. Eng.* 2018. 10.1115/1.4040019. 29801168 10.1115/1.4040019

[25] Bland, M. L., C. McNally, D. S. Zuby, B. C. Mueller, and S. Rowson. Development of the STAR evaluation system for assessing bicycle helmet protective performance. *Ann. Biomed. Eng.* 48(1):47–57, 2020. 10.1007/s10439-019-02330-0. 31372859 10.1007/s10439-019-02330-0 PMC6928078

[26] Bliven, E., et al. Evaluation of a novel bicycle helmet concept in oblique impact testing. *Accid. Anal. Prev.* 124:58–65, 2019. 10.1016/j.aap.2018.12.017. 30634159 10.1016/j.aap.2018.12.017 PMC6743977

[27] Bonin, S. J., A. L. DeMarco, and G. P. Siegmund. The effect of MIPS, headform condition, and impact orientation on headform kinematics across a range of impact speeds during oblique bicycle helmet impacts. *Ann. Biomed. Eng.* 50(7):860–870, 2022. 10.1007/s10439-022-02961-w. 35441268 10.1007/s10439-022-02961-w

[28] Stark, N.E.-P., C. Clark, and S. Rowson. Human head and helmet interface friction coefficients with biological sex and hair property comparisons. *Ann. Biomed. Eng.* 2023. 10.1007/s10439-023-03332-9. 37540293 10.1007/s10439-023-03332-9 PMC11402834

[29] N. Stark, T. Geiman, S. Gagliardi, M. Wood, L. Viano, and S. Rowson, “Headform Friction Coefficients Relevant to Helmet Testing,” *IRCOBI*, pp. 956–967, 2023.

[30] Instrumentation for Impact Test, SAE J211, Aug. 19, 2022.

[31] Calculation Guidelines for Impact Testing, SAE J1727, Oct. 29, 2021.

[32] “Safety for helmets,” Mips. Accessed: Jan. 28, 2025. [Online]. Available: https://mipsprotection.com/

[33] “Technology – WaveCel.” Accessed: Jan. 28, 2025. [Online]. Available: https://wavecel.com/technology/

[34] “Lazer’s New Proprietary Integrated Rotational Impact Protection,” Lazer Sport. Accessed: Jan. 28, 2025. [Online]. Available: https://lazersport.us/pages/kineticore

[35] Kuznetsova, A., P. B. Brockhoff, and R. H. B. Christensen. lmerTest package: tests in linear mixed effects models. *J. Stat. Softw.* 82:1–26, 2017. 10.18637/jss.v082.i13.

[36] ASTM F1492-22 - Standard Specification for Helmets Used in Skateboarding and Trick Roller Skating, F1492-22, Jul. 22, 2022. [Online]. Available: https://compass.astm.org/document/?contentCode=ASTM%7CF1492-22%7Cen-US&proxycl=https%3A%2F%2Fsecure.astm.org&fromLogin=true

[37] Stark, N., S. Piwowarski, A. Calis, M. Wood, M. Begonia, and S. Rowson, “Surface Friction Implications for Snowsport Helmets,” *IRCOBI*, pp. 460–471, 2024.

[38] Bland, M. L., et al. Laboratory reconstructions of bicycle helmet damage: investigation of cyclist head impacts using oblique impacts and computed tomography. *Ann. Biomed. Eng.* 48(12):2783–2795, 2020. 10.1007/s10439-020-02620-y. 32974755 10.1007/s10439-020-02620-y

